# Isoprene nitrates drive new particle formation in Amazon’s upper troposphere

**DOI:** 10.1038/s41586-024-08192-4

**Published:** 2024-12-04

**Authors:** Joachim Curtius, Martin Heinritzi, Lisa J. Beck, Mira L. Pöhlker, Nidhi Tripathi, Bianca E. Krumm, Philip Holzbeck, Clara M. Nussbaumer, Lianet Hernández Pardo, Thomas Klimach, Konstantinos Barmpounis, Simone T. Andersen, Roman Bardakov, Birger Bohn, Micael A. Cecchini, Jean-Pierre Chaboureau, Thibaut Dauhut, Dirk Dienhart, Raphael Dörich, Achim Edtbauer, Andreas Giez, Antonia Hartmann, Bruna A. Holanda, Philipp Joppe, Katharina Kaiser, Timo Keber, Hannah Klebach, Ovid O. Krüger, Andreas Kürten, Christian Mallaun, Daniel Marno, Monica Martinez, Carolina Monteiro, Carolina Nelson, Linda Ort, Subha S. Raj, Sarah Richter, Akima Ringsdorf, Fabio Rocha, Mario Simon, Sreedev Sreekumar, Anywhere Tsokankunku, Gabriela R. Unfer, Isabella D. Valenti, Nijing Wang, Andreas Zahn, Marcel Zauner-Wieczorek, Rachel I. Albrecht, Meinrat O. Andreae, Paulo Artaxo, John N. Crowley, Horst Fischer, Hartwig Harder, Dirceu L. Herdies, Luiz A. T. Machado, Christopher Pöhlker, Ulrich Pöschl, Anna Possner, Andrea Pozzer, Johannes Schneider, Jonathan Williams, Jos Lelieveld

**Affiliations:** 1 Institute for Atmospheric and Environmental Sciences, Goethe University Frankfurt, Frankfurt am Main, Germany; 2 Atmospheric Microphysics Department, Leibniz Institute for Tropospheric Research, Leipzig, Germany; 3 Faculty of Physics and Earth Sciences, Leipzig Institute for Meteorology, Leipzig University, Leipzig, Germany; 4 Max Planck Institute for Chemistry, Mainz, Germany; 5 Lemon Labs Ltd., Nicosia, Cyprus; 6 Department of Environmental Science, Stockholm University, Stockholm, Sweden; 7 Bolin Centre for Climate Research, Stockholm University, Stockholm, Sweden; 8 Institute of Climate and Energy Systems (ICE-3), Forschungszentrum Jülich GmbH, Jülich, Germany; 9 Institute of Astronomy, Geophysics and Atmospheric Sciences, University of São Paulo, São Paulo, Brazil; 10 Laboratoire d’Aérologie, Université de Toulouse, CNRS, UT3, IRD, Toulouse, France; 11 Flight Experiments, German Aerospace Center (DLR), Weßling, Germany; 12 Institute for Atmospheric Physics, Johannes Gutenberg-University, Mainz, Germany; 13 National Institute for Space Research, Cachoeira Paulista, Brazil; 14 National Institute of Amazonian Research, Manaus, Brazil; 15 Institute of Meteorology and Climate Research (IMK), Karlsruhe Institute of Technology (KIT), Eggenstein-Leopoldshafen, Germany; 16 Department of Geology and Geophysics, King Saud University, Riyadh, Saudi Arabia; 17 Scripps Institution of Oceanography, University of California, San Diego, La Jolla, CA USA; 18 Center for Sustainable Amazon Studies (CEAS), University of São Paulo, São Paulo, Brazil; 19 Instituto de Física, University of São Paulo, São Paulo, Brazil; 20 Climate and Atmosphere Research Center, The Cyprus Institute, Nicosia, Cyprus

**Keywords:** Atmospheric chemistry, Climate sciences, Atmospheric chemistry

## Abstract

New particle formation (NPF) in the tropical upper troposphere is a globally important source of atmospheric aerosols^1–4^. It is known to occur over the Amazon basin, but the nucleation mechanism and chemical precursors have yet to be identified^2^. Here we present comprehensive in situ aircraft measurements showing that extremely low-volatile oxidation products of isoprene, particularly certain organonitrates, drive NPF in the Amazonian upper troposphere. The organonitrates originate from OH-initiated oxidation of isoprene from forest emissions in the presence of nitrogen oxides from lightning. Nucleation bursts start about 2 h after sunrise in the outflow of nocturnal deep convection, producing high aerosol concentrations of more than 50,000 particles cm^−^^3^. We report measurements of characteristic diurnal cycles of precursor gases and particles. Our observations show that the interplay between biogenic isoprene, deep tropical convection with associated lightning, oxidation photochemistry and the low ambient temperature uniquely promotes NPF. The particles grow over time, undergo long-range transport and descend through subsidence to the lower troposphere, in which they can serve as cloud condensation nuclei (CCN) that influence the Earth’s hydrological cycle, radiation budget and climate^1,4–8^.

## Abstract (web summary)

Aircraft measurements over the Amazon show that new particle formation in the upper troposphere emerges when isoprene, emitted by forests, undergoes oxidation in the presence of nitrogen oxides produced by lightning.

## Main

Isoprene is emitted in large quantities by vegetation such as broad-leafed trees and it has the largest source strength of all biogenic volatile organic compounds (VOCs). Globally, emissions amount to about 500–600 Tg a^−1^, representing more than half of the total biogenic VOC emissions, with tropical South America contributing an estimated 163 Tg a^−1^ (refs. ^9,10^). Isoprene is primarily emitted into the atmosphere during daylight hours and is typically converted within 1–2 h into oxygenated VOCs, mostly through reaction with hydroxyl radicals (OH)^11,12^. Isoprene mixing ratios in the continental boundary layer of the Amazon basin range between 1 and 20 ppbv, with a diurnal cycle peaking in the afternoon^13^. Although isoprene-derived oxygenated organic molecules (IP-OOMs) are known to contribute substantially to the production of secondary organic aerosol mass by means of condensation onto pre-existing particles^14,15^, they are unable to form new particles in the boundary layer and are even thought to inhibit NPF from monoterpenes^16,17^.

Deep convection can transport isoprene from the boundary layer to the upper troposphere within 1–2 h (refs. ^18,19^). Palmer et al.^20^ demonstrated that nocturnal deep convection delivers isoprene to the tropical upper troposphere, leading to secondary organic aerosol production^20^. Deep convective clouds are also associated with lightning activity^21^ and thus represent a main source of nitrogen oxides (NO_*x*_)^22^ that play a central role in OH formation (recycling) in the upper troposphere^23^. Furthermore, pre-existing aerosol particles are efficiently scavenged from boundary-layer air that ascends in precipitating deep convective clouds^2,24^. As a result, the outflow of nocturnal deep convection contains increased levels of isoprene and NO_*x*_, with a weak condensation sink (CS) for condensation of vapours onto pre-existing aerosols. The low temperatures of the upper troposphere and intense solar (actinic) radiation during the daytime create favourable conditions for NPF, that is, aerosol nucleation and initial growth up to detectable particle size in the convective outflow^1,2,7,25^.

Although high number concentrations of freshly formed particles have frequently been observed in the upper troposphere^1–3,7,25–28^, the conditions and chemical processes underlying NPF have remained elusive. Kulmala et al.^29^ proposed that insoluble organics are transported to the upper troposphere by deep convection to produce new particles and Ekman et al.^30^ suggested that isoprene itself could be the precursor for upper tropospheric NPF over the Amazon. A study of the aerosol composition in the upper troposphere over the Amazon, focusing on larger particles (*d*_p_ > 50 nm), found isoprene-derived secondary organic aerosols and organic nitrates to be the main components^31^. Recent measurements at the Chacaltaya mountain site in Bolivia (5,240 m a.m.s.l.) revealed isoprene oxidation products, including organonitrates in the gas and aerosol phases in air masses from the free troposphere over the Amazon basin^32^. However, the precursors that drive particle nucleation and early growth were not identified. Although NPF supplies about half the CCN globally^4,33^, in the continental and marine boundary layer in the tropics it is rare^34^ and typical NPF events have not been observed over the Amazon rainforest, mostly because vapours that are condensable at cold conditions remain volatile at high temperatures^35–38^. However, several studies have demonstrated that particles form in the cold upper troposphere and are subsequently transported to lower altitudes, at which they act as CCN^1,2,4,6,7,39,40^.

## Observation of NPF

**Fig. 1:**
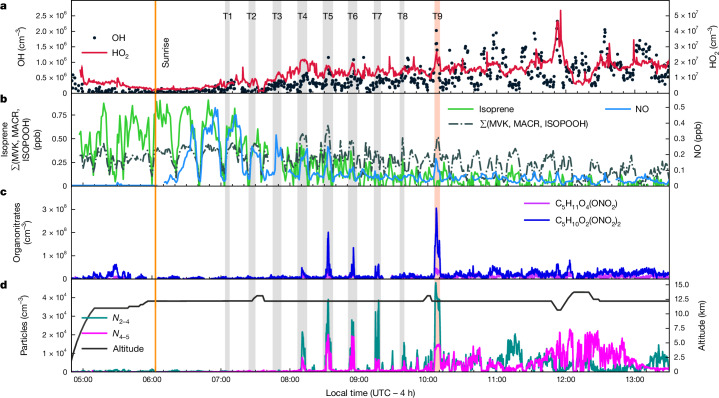
Time series of radicals, trace gases and aerosol particles. Measurements were performed on 23 January 2023 over the Amazon basin north of Manaus (RF 19). **a**, OH and HO_2_. **b**, Isoprene, sum of the first-generation isoprene oxidation products methyl vinyl ketone (MVK), methacrolein (MACR) and isoprene hydroxy hydroperoxide (ISOPOOH) and NO. **c**, Isoprene-derived organonitrates C_5_H_11_O_4_(ONO_2_) and C_5_H_10_O_2_(ONO_2_)_2_. **d**, Particle concentrations *N*_2–4_ and *N*_4–5_, as well as pressure altitude. The light-red bar (T9) indicates the period between 10:05 and 10:10 local time, during which the strongest NPF was detected. Grey bars (T1–T8) represent previous sampling periods of the air mass examined at T9, traced by backward trajectories originating from the position of the aircraft during that period. The ambient temperature was about −58 °C at a flight altitude of 12.3 km during T1–T9. During the 4–5 min of the NPF event encounters, the aircraft travels more than 50 km. Source Data

**Fig. 2:**
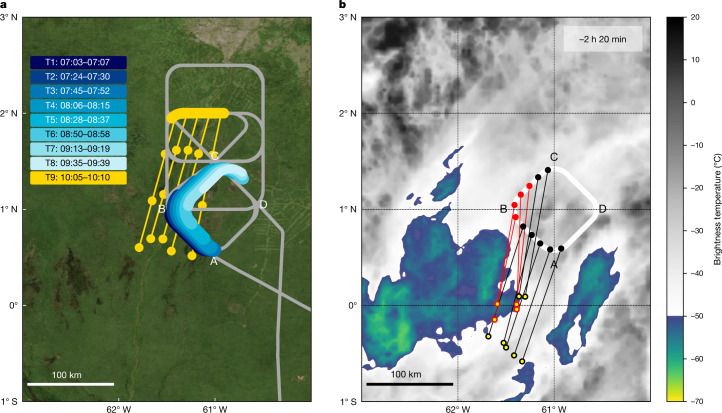
Flight path and air-mass back trajectories. **a**, Flight path for RF 19 (grey). The diamond-shaped pattern ABCD was flown repeatedly and the NPF air mass was encountered several times (thick-coloured sections T4–T9) also before the NPF event started (T1–T3). Coloured sections T1–T8 are each slightly shifted to the right to prevent overlap. Thin yellow lines with 1-h markers indicate 3 h of back trajectories from the NPF period T9 used to calculate and identify periods T1–T8. **b**, Back trajectories from the locations of the aircraft between 08:05 and 08:15 local time (black and red markers) to 140 min before (yellow-centred markers) and infrared satellite image (GOES-16, band 13: 10.3 µm; https://ftp.cptec.inpe.br/goes/goes16/retangular/ch13/2023/01/) indicating the approximate cloud-top temperatures at 05:50 local time. Temperatures below −50 °C are coloured. The back-trajectory calculations used the horizontal wind speed and direction measured by the research aircraft. The air parcels in which NPF was detected (red circles) trace back to the convective cloud and were in contact with convection more recently (relative to the sampling time) than those in which NPF was not detected (black circles). Satellite picture data in **a** obtained from https://wvs.earthdata.nasa.gov, NASA Worldview Snapshots. Source Data

**Extended Data Fig. 1:**
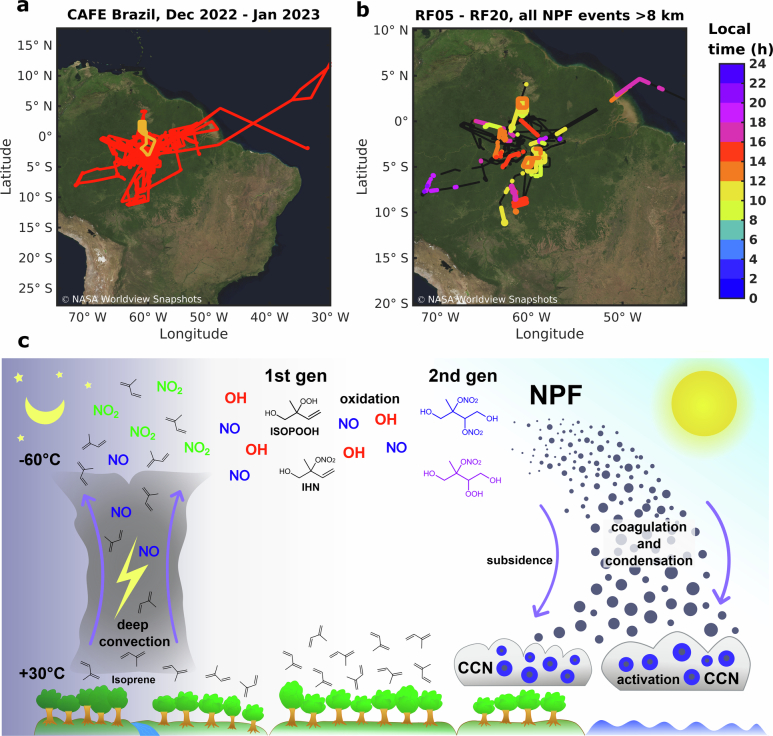
Overview of flight tracks, NPF events and schematic of the isoprene-driven NPF. **a**, HALO aircraft flight tracks during CAFE-Brazil. RF 19, described in detail in this study, is highlighted in orange. **b**, NPF events over the Amazon basin and adjacent areas. Flight path segments are shown as black lines for flight altitudes >8 km for all research flights RF 05 to RF 20 in Brazil (except RF 16, and for RF 17, channel *N*_2_ is replaced by channel *N*_3_). NPF data points are coloured according to the local time of measurement. **c**, Transport and chemistry of isoprene-driven NPF. Emission of isoprene by vegetation, transport to the upper troposphere by nocturnal deep convection, photochemical formation of first-generation and second-generation oxidation products owing to reactions involving OH and NO at upper tropospheric temperatures, NPF from isoprene-derived organonitrates, zonal and downward transport, aerosol growth to CCN size and activation of CCN into cloud droplets. Satellite picture data in **a** and **b** from https://wvs.earthdata.nasa.gov. Source Data

**Extended Data Fig. 2:**
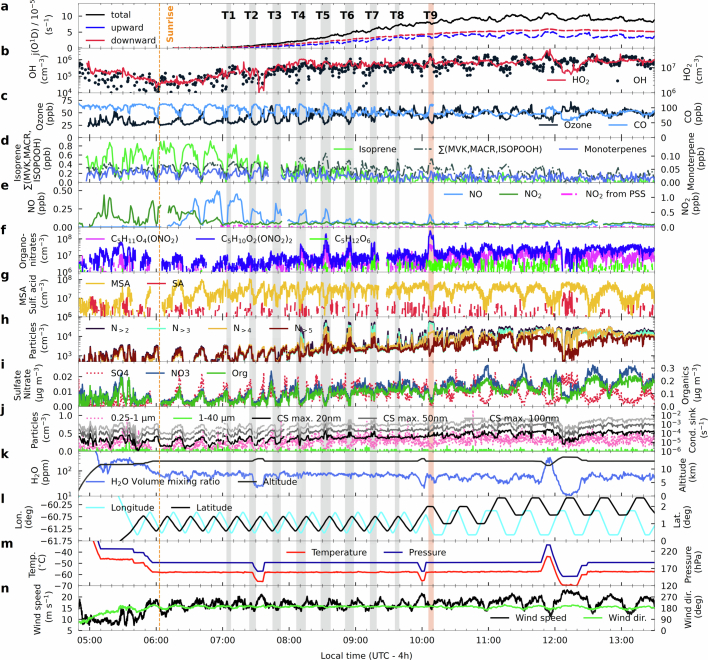
Extended dataset for RF 19. **a**, Actinic flux *j*(O^1^D) resulting from total spectral actinic flux densities (downward + upward). Note that the high reflectivity from a cirrus cloud deck below the aircraft increased the total flux considerably. **b**, OH and HO_2_. **c**, Ozone and CO. **d**, isoprene, sum of methyl vinyl ketone (MVK), methacrolein (MACR) and isoprene hydroxyhydroperoxide (ISOPOOH), monoterpenes. **e**, NO, NO_2_ (upper limit) and NO_2_ calculated from photostationary state using NO, O_3_ and *j*(NO_2_). **f**, Organonitrates C_5_H_11_O_4_(ONO_2_) and C_5_H_10_O_2_(ONO_2_)_2_. **g**, Gaseous sulfuric acid and MSA (upper limit). Note that a fraction of the signals (especially for methane sulfonic acid) is probably because of evaporation of pre-existing particles in the inlet line. **h**, Particle number concentrations *N*_2_, *N*_3_, *N*_4_ and *N*_5_. **i**, Aerosol compounds sulfate, nitrate and organics for particles >50 nm (AMS measurements). Note the high correlation between organics and nitrate also for the particles >50 nm. **j**, Particle concentration for particle sizes 0.25–1 µm and 1–40 µm (OPC measurements) and estimated CS for different assumptions of particle size. **k**, Water vapour and flight altitude. **l**, Latitude and longitude of aircraft position. **m**, Ambient temperature and pressure. **n**, Wind velocity and direction. For details on uncertainties and detection limits, see Methods. Source Data

The Chemistry of the Atmosphere: Field Experiment in Brazil (CAFE-Brazil) took place from December 2022 to January 2023 (Extended Data Fig. 1). It studied NPF by conducting several measurement flights in the upper troposphere up to 13.8 km altitude over the Amazon basin (Methods). High concentrations of freshly formed aerosol particles (*d*_p_ < 5 nm) were frequently detected at altitudes above 8 km. Several condensation nuclei (CN)-counter channels were operated to measure the total particle number concentrations *N*_2_, *N*_3_, *N*_4_ and *N*_5_ larger than the lower detection cut-off diameters of 2, 3, 4 and 5 nm, respectively, and the differences, for example, *N*_2–5_, denote the concentration of freshly formed particles in the range 2–5 nm (Methods). In situ observations of the chemical transformation of precursor gases into extremely low-volatility organic compounds (ELVOCs) and the onset of rapid and strong NPF in air masses influenced by recent outflow from a nocturnal mesoscale convective system are shown in Fig. 1. By repeatedly examining the same air masses while flying a holding pattern centred on the outflow, we followed the photochemical development during the night–day transition (Extended Data Fig. 2). Measurements before sunrise were used to characterize air masses at an altitude of 13 km. Higher isoprene mixing ratios of 400–850 pptv (equivalent to pmol mol^−1^) were found up to 3 h after cloud outflow. Trajectory calculations indicate that the air had been in contact with an electrically active mesoscale convective system that had developed south of the flight path during night-time (Fig. 2), which was sampled frequently (T1–T9 in Figs. 1 and 2). The outflow also comprised high levels of NO_*x*_. Photochemical production of OH radicals was observed to start at sunrise and increased with solar elevation, reaching concentrations of about 6 × 10^5^ cm^−3^ (corresponding to 0.1 pptv) during T4. During this period, radiation reflected from cirrus layers below the sampled air masses resulted in an increase in actinic flux and enhanced rates of photochemical processing, with 300–500 pptv of NO formed from NO_2_ photolysis after sunrise (Fig. 1 and Extended Data Fig. 2). The reaction of isoprene with OH initiates the formation of isoprene hydroxy peroxy radicals (ISOPOO).

**Fig. 3:**
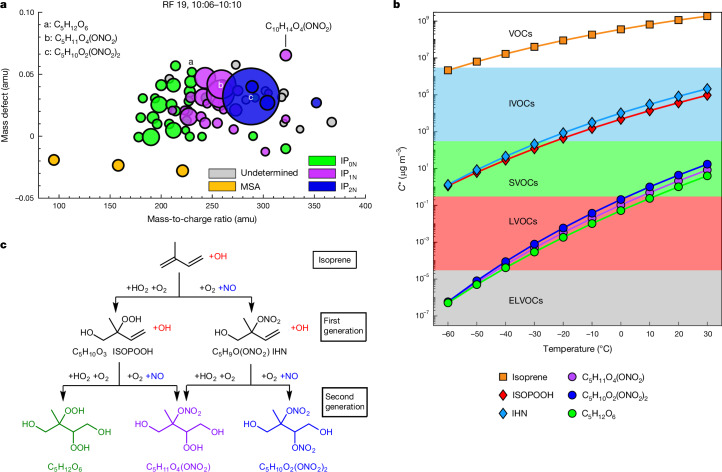
Molecular composition detected with the CI-APi-TOF mass spectrometer during the strongest NPF event of RF 19 (T9, 10:06–10:10 local time). **a**, Mass defect versus mass-to-charge ratio with labelling of the molecular composition for prominent peaks. For clarity, the nitrate reagent ion (NO_3_^−^, *m*/*z* = 62) has been omitted from all labels. Circle size indicates peak intensity and colour indicates isoprene oxidation products without a nitrate group (IP_0N,_ green), mononitrates (IP_1N_, magenta), dinitrates (IP_2N_, blue) and MSA signals (orange). **b**, Saturation concentration C* of isoprene and its oxidation products as calculated with the SIMPOL model^49^. The compounds C_5_H_12_O_6_, C_5_H_11_O_4_(ONO_2_) and C_5_H_10_O_2_(ONO_2_)_2_ are ELVOCs at −58 °C. **c**, Reaction scheme leading to the formation of C_5_H_12_O_6_, C_5_H_11_O_4_(ONO_2_) and C_5_H_10_O_2_(ONO_2_)_2_. The molecular structures shown are exemplary. IVOCs, intermediate-volatility organic compounds; LVOCs, low-volatility organic compounds; SVOCs, semivolatile organic compounds. Source Data

**Extended Data Fig. 3:**
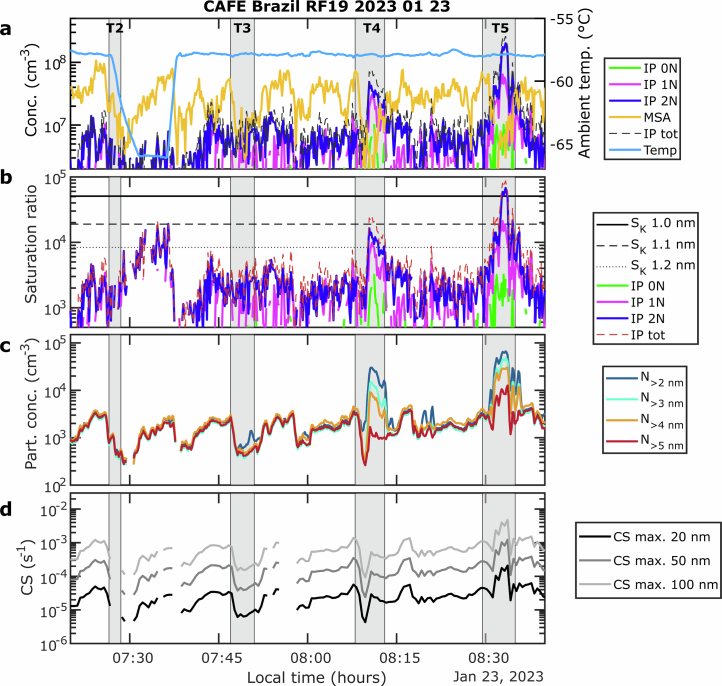
Details of NPF event during RF 19. **a**, IP_0N_, IP_1N_ and IP_2N_ measured with the CI-APi-TOF mass spectrometer as well as IP_0-2N_ = IP_0N_ + IP_1N_ + IP_2N_ and MSA. **b**, Calculated saturation ratio for IP_0N_, IP_1N_ and IP_2N_, as well as estimated saturation ratio necessary to overcome the Kelvin barrier for particle diameters of 1.0, 1.1 and 1.2 nm, respectively (Methods). **c**, Particle concentrations *N*_2_, *N*_3_, *N*_4_ and *N*_5_ as measured by four channels of the FASD instrument. **d**, Estimated maximum of the CS when assuming all particles *N*_5_ to have maximum sizes of 20, 50 or 100 nm. During T3, when NPF is first observed by the initial increase of *N*_2_, the CS is less than 2 × 10^−4^ s^−1^. Source Data

**Extended Data Fig. 4:**
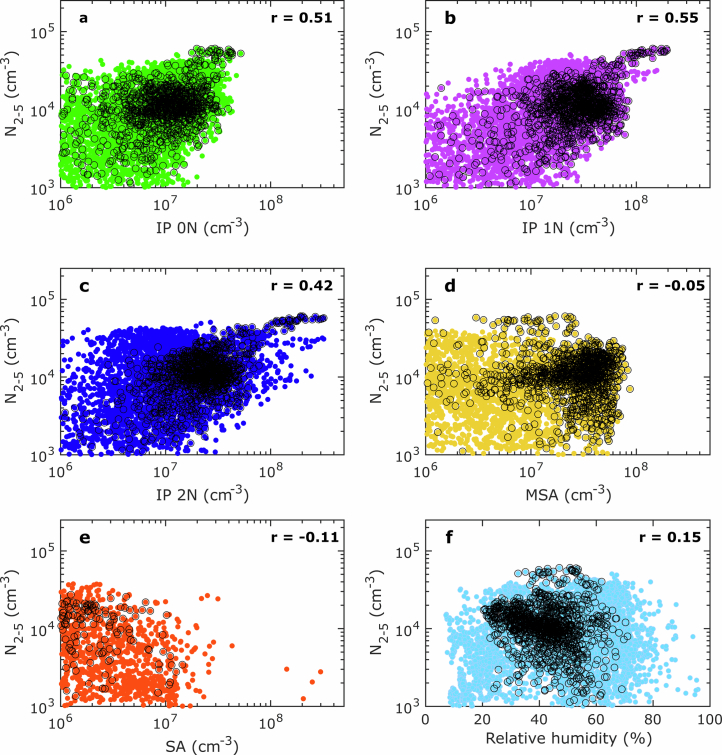
Correlation of *N*_2–5_ particle concentration with trace gases. Correlation of *N*_2–5_ with IP_0N_ (**a**), IP_1N_ (**b**), IP_2N_ (**c**), MSA (**d**), sulfuric acid (**e**) and relative humidity (**f**). Coloured data represent research flights RF 09 to RF 20, excluding RF 16, and for RF 17, channel *N*_2_ is replaced by *N*_3_ for technical reasons. Only data during NPF events measured between 08:00 and 18:00 local time (LT) and for altitudes >8 km are shown (see Fig. 4 and Extended Data Fig. 8). Data points with black circles represent RF 19. Correlation coefficients represent the entire dataset. Source Data

**Extended Data Fig. 5:**
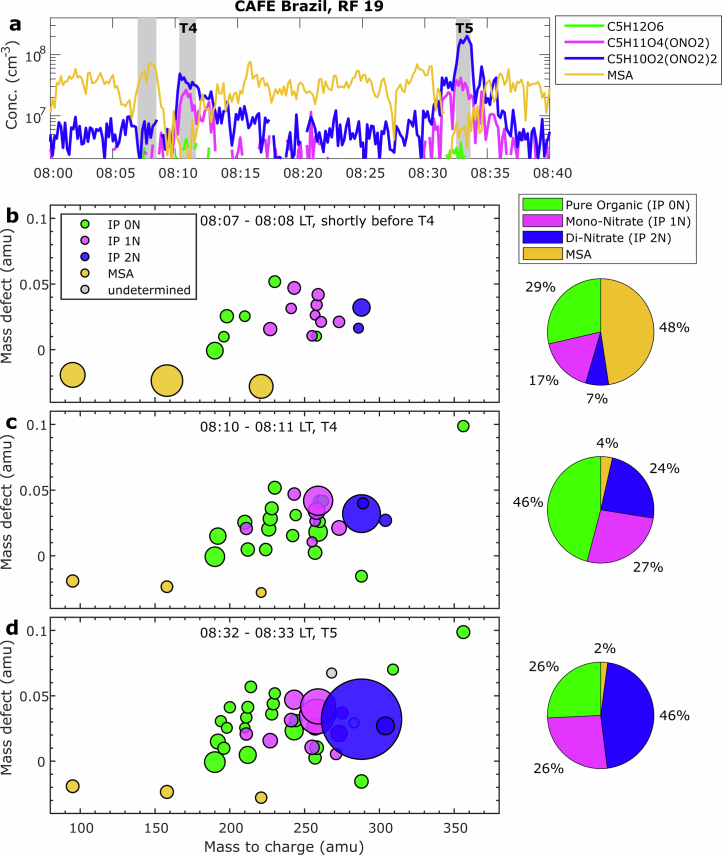
Time series and mass-defect plots for CI-APi-TOF data before and during NPF events of RF 19. **a**, Time series of the most prominent mononitrates and dinitrates C_5_H_11_O_4_(ONO_2_) and C_5_H_10_O_2_(ONO_2_)_2_, representative IP_0N_ compound C_5_H_12_O_6_ and MSA. **b**, Signals strongly shift between an air parcel that is not influenced by the fresh convection (which contains larger amounts of pre-existing particles that probably contribute to the MSA signal owing to particle evaporation in the inlet) and signals during the first (**c**) and second (**d**) encounters of the strong NPF event (T4 and T5). High concentrations of the mononitrates and dinitrates are rapidly forming. Source Data

Further reactions lead to forming first-generation and second-generation products, including various organonitrates. High concentrations of highly oxidized isoprene alkyl nitrates were observed for the first time at 08:10–08:14 local time (T4), coincident with bursts of *N*_2–5_ particles (Fig. 1 and Extended Data Figs. 2–5). The chemical ionization atmospheric pressure interface ime-of-flight (CI-APi-TOF) mass spectrometer can detect highly oxidized ELVOCs as well as inorganic species such as sulfuric acid^41–43^, which cause NPF (Methods). The CI-APi-TOF data were strongly dominated by the mononitrate C_5_H_11_O_4_(ONO_2_) and the dinitrate C_5_H_10_O_2_(ONO_2_)_2_ of isoprene (Fig. 3), causing nucleation and the early growth of the freshly formed particles (Methods and Extended Data Fig. 3). These molecular properties have been confirmed by laboratory observations of isoprene nitrates in charged molecular clusters and neutral particles at upper-troposphere conditions^44^. The CI-APi-TOF measurements represent a combination of gas phase and nanoaerosol composition owing to ram-pressure-induced adiabatic heating and, thus, partial evaporation of molecular clusters and nanoparticles in the inlet system (Methods).

The air mass in which the first NPF burst was observed at 08:10–08:14 local time (T4) initially contained a low number of pre-existing particles (*N*_2_ < 3,000 cm^−3^) and hence a low CS, estimated at CS < 1 × 10^−^^3^ s^−1^ (T2 and T3; Methods and Extended Data Fig. 3). Although no *N*_2–5_ particles were detected in T2, several hundred more particles were detected in the 2-nm CN-counter channel during period T3, indicating the onset of NPF (Extended Data Fig. 3). Within the 20 min between T3 and T4, more than 25,000 cm^−^^3^ *N*_2–5_ particles were formed, which corresponds to a formation rate *J*_2_ for particles >2 nm of about 20 cm^−3^ s^−1^. For this first NPF burst, particle concentrations measured in all CN-counter channels increased by at least an order of magnitude, except for the largest channel (>5 nm), which only increased slightly over previous background values. This implies a growth rate of about 9 nm h^−1^ from an initial cluster size of around 1 nm up to 4 nm. At the subsequent encounter of the air mass, another 20 min later (T5), the 5-nm channel shows an increase by one order of magnitude, indicating growth of the 2–5-nm particles to sizes beyond 5 nm. The growth rate represents an estimate, limited by the uncertainties of cut-off diameters of the CN-counter channels and air-mass inhomogeneities. Later in the day, *N*_2–5_ particles were ubiquitous throughout the upper troposphere, indicating that NPF events are widespread, and nucleation plumes disperse and mix with background air. At that time, the numbers of *N*_2–5_ particles did not reach the same levels as during the early morning, indicating reduced availability of precursors and particle abundance owing to coagulation and mixing. During the later flight sections, we could not repeat quasi-Lagrangian air-mass encounters, thus formation and growth rates cannot be estimated. Nevertheless, the CN-counter measurements confirm the presence of *N*_2–5_ particles.

## Formation of organonitrates

The highest concentrations of nitrates and *N*_2–5_ particles were detected at 10:06–10:10 local time (T9), for which CI-APi-TOF data are shown in Fig. 3. Isoprene oxygenated organic molecules comprise about 97% of the detected reaction products, with two compounds dominating, C_5_H_11_O_4_(ONO_2_) and C_5_H_10_O_2_(ONO_2_)_2_. We categorized all isoprene oxidation products detected by the CI-APi-TOF mass spectrometer into non-nitrogen-containing, mononitrates and dinitrates (IP_0-2N_ = IP_0N_ + IP_1N_ + IP_2N_). The CI-APi-TOF mass spectrometer only detects oxidized isoprene products with number of oxygen atoms ≥ 4 and with increasing sensitivity with the number of oxygen atoms and functional groups^41^ (Methods). Therefore, these measurements represent lower limits of the ambient concentrations of compounds that contain 4–9 oxygen atoms and, although species such as C_5_H_8_O_4_ are detected, they may be underestimated.

The two main compounds C_5_H_11_O_4_(ONO_2_) and C_5_H_10_O_2_(ONO_2_)_2_ are the likely products of second-generation OH oxidation in which the intermediate RO_2_ radicals react twice (sequentially) with NO to form the dinitrate C_5_H_10_O_2_(ONO_2_)_2_ or with HO_2_ and NO to form the mononitrate C_5_H_11_O_4_(ONO_2_), respectively (Fig. 3c). The corresponding first-generation intermediate closed-shell products are isoprene hydroxyl peroxide (ISOPOOH, C_5_H_10_O_3_), formed when ISOPOO reacts with HO_2_, and isoprene hydroxy nitrate (IHN, C_5_H_9_O(ONO_2_)) in the case of NO reaction. The isomeric structures of the first-generation and second-generation products shown in Fig. 3c are exemplary; other formation pathways may exist and further reaction pathways (for example, forming peroxy nitrates RO_2_NO_2_ from reaction with NO_2_) complement the scheme. Although isoprene oxidation chemistry has been investigated in detail, including under high-NO_*x*_ conditions^11,45–48^, studies at low-temperature and low-pressure conditions in the upper troposphere are lacking. The reaction of NO with the RO_2_ peroxy radical can produce either nitrates or alkoxy radicals and NO_2_. The branching ratio for this reaction is an intricate function of temperature, pressure, molecular size and structure. Still, at the low-temperature conditions in the upper troposphere, the branching ratio probably shifts towards the termolecular channel, which produces organic nitrates^11^.

Further isoprene nitrate compounds include, for example, C_5_H_11_NO_8_ and C_5_H_10_N_2_O_9_, which are probably peroxynitrates formed from the reaction of the RO_2_ radicals with NO_2_. These compounds were also detected, but at 10–40 times lower abundance compared with the respective alkyl nitrates (Fig. 3). The lower abundance is explained by the much lower concentration of NO_2_ (as derived from the photostationary state approximation) compared with NO (Extended Data Fig. 2).

We used the SIMPOL model^49^ to estimate the volatility and the supersaturation ratio for C_5_H_12_O_6_, C_5_H_11_O_4_(ONO_2_) and C_5_H_10_O_2_(ONO_2_)_2_ (Methods). At −60 °C in the upper troposphere, all three compounds have a saturation concentration, C*, of about 3 × 10^−7^ µg m^−3^, which is in the volatility class of ELVOCs^50^. At T9, a total supersaturation ratio of 0.5–1.0 × 10^5^ is calculated. For these conditions, C_5_H_11_O_4_(ONO_2_) and C_5_H_10_O_2_(ONO_2_)_2_ are expected to participate in particle nucleation and early growth. It can be expected that the nucleation is boosted by the involvement of IP_0N_, as well as small amounts of inorganic species, such as sulfuric acid^44^. The influence of H_2_SO_4_ on the nucleation cannot be directly inferred from our observations as the sulfuric acid concentration was low, below the detection limit of about 2.0 × 10^6^ molecules cm^−^^3^ (Methods).

## Role of monoterpenes and ions

Highly oxidized organic molecules (HOMs) generated from the oxidation of monoterpenes such as α-pinene have been suggested to cause NPF in the tropical upper troposphere owing to their extremely low or ultralow volatility^42,43^. Our observations at high altitudes show no evidence of monoterpene-derived HOMs (neither nitrates nor non-nitrates) and therefore do not support this hypothesis. We only observed typical monoterpene-derived HOMs in the Amazon boundary layer. Even in fresh convective outflow, the monoterpene mixing ratio in the upper troposphere was typically well below 60 pptv (Extended Data Fig. 2), providing insufficient precursor concentrations to explain our NPF data. Furthermore, HOM formation in the upper troposphere is too slow^43^ for monoterpenes to play an important role in NPF or early particle growth, as the HOM yield decreases with decreasing temperature.

Ion-induced nucleation^51,52^ could play a notable role in NPF, in which ions produced from galactic cosmic rays may promote IP-OOMs nucleation^44^. This nucleation pathway is limited by the ion pair production rate in the tropical upper troposphere, which may reach 40 cm^−3^ s^−1^ (ref. ^51^). A NPF rate of about 20 cm^−3^ s^−^^1^, as determined by our observations, is thus within this range. Nevertheless, our observations show that the limiting factor for NPF is not the presence of ions (which are ubiquitous) but, rather, is determined by the photochemical generation of ELVOCs from isoprene.

## Diel cycles of NPF

**Fig. 4:**
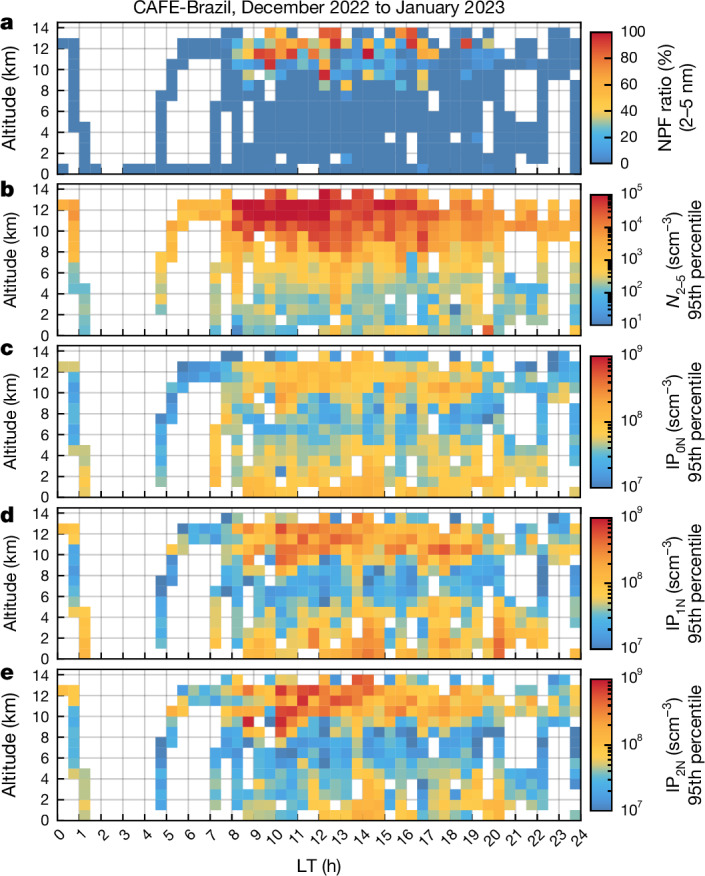
Diurnal cycle and altitude dependence of NPF events, *N*_2–5_ particles and precursor gases. Pixels are calculated as bins of 1 km altitude and 30 min time and combine data from 11 research flights over the Amazon basin. **a**, Frequency of occurrence of NPF events, defined as the ratio of NPF events to the total number of measurements for each pixel. **b**, Concentrations of *N*_2–5_ particles. **c**–**e**, Isoprene organonitrates IP_0N_ (**c**), mononitrates IP_1N_ (**d**) and dinitrates IP_2N_ (**e**). Panels **b–e** show the 95th percentile of the concentration, that is, 5% of the data contributing to a pixel are even higher than the value indicated by the colour. In the upper troposphere, high concentrations of organonitrates and *N*_2–5_ particles occurred frequently, whereas concentrations were low in the lower and middle tropospheres and during the night. Data influenced by the Manaus plume and by biomass burning have been removed. For direct comparability, all concentration data are normalized to standard conditions (273.15 K, 1,000 hPa). Source Data

**Extended Data Fig. 6:**
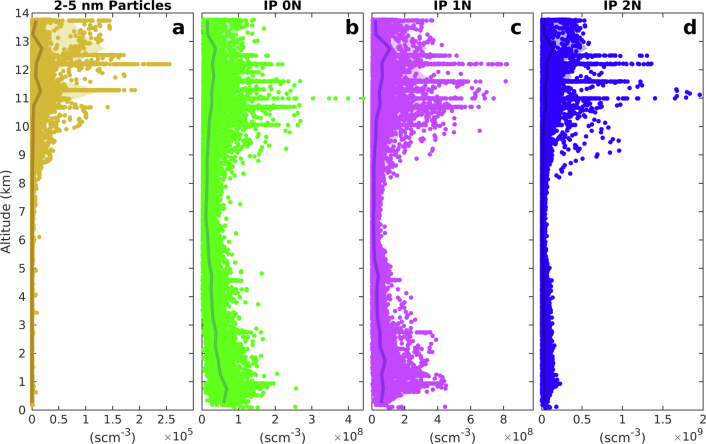
Altitude profiles for *N*_2–5_ particles and isoprene oxidation products IP_0,1,2N_ measured by the CI-APi-TOF mass spectrometer. All data were converted to standard conditions (273.15 K, 1,000 hPa) for altitude-independent comparability and comparability with previous studies^1,2^. Converted to standard conditions, maximum particle concentrations *N*_2–5_ for upper tropospheric NPF events were frequently larger than 100,000 scm^−3^. Data for flights RF 09 to RF 20, excluding RF 16 and using *N*_3–5_ for RF 17. Data influenced by biomass burning and by the Manaus plume have been removed. Grey lines denote average concentrations and shaded areas give 5th to 95th percentiles. Source Data

**Extended Data Fig. 7:**
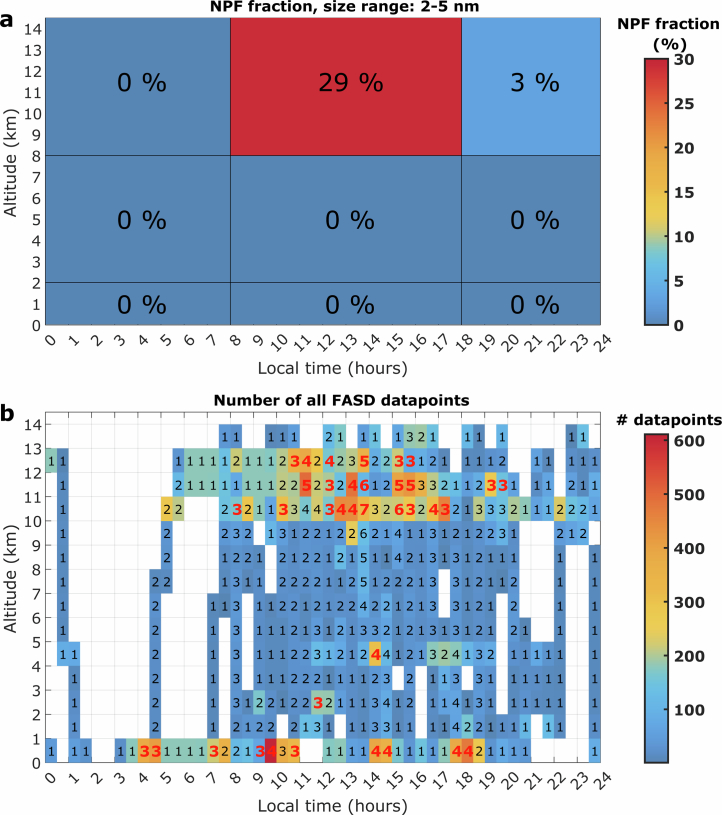
Frequency of NPF events detected during RF 09 to RF 20. **a**, Data from Fig. 4 are binned vertically into boundary layer (0–2 km), lower and middle free troposphere (2–8 km) and the upper troposphere (>8 km). Time bins with respect to day-time when NPF is observed (08:00–18:00 LT) as well as night-time and early morning (before 08:00 LT and after 18:00 LT). **b**, The number of measurements per pixel for the data shown in Fig. 4. The number in each pixel denotes the number of contributing aircraft flights. Numbers in red denote pixels with at least three flights and 20 data points per flight. The colours denote the total number of data points obtained, with each data point representing a 1-min average value during a flight. Data from RF 16 are excluded; *N*_2–5_ is replaced by *N*_3–5_ for RF 17. Source Data

**Extended Data Fig. 8:**
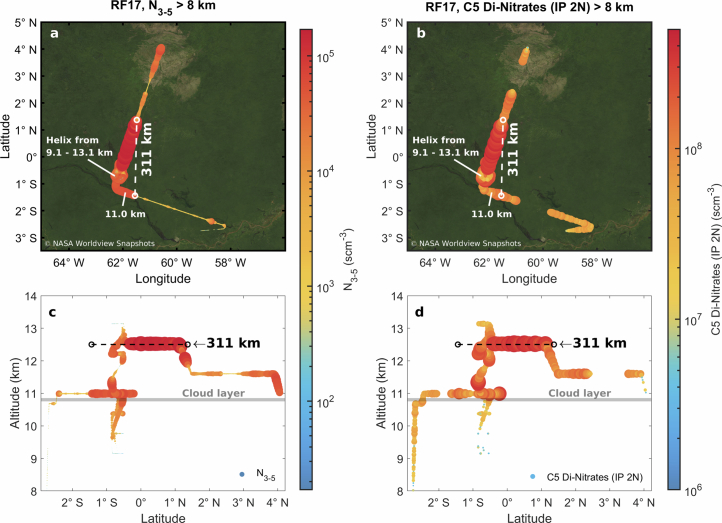
Spatial extent of the NPF event observed during RF 17, measured in the morning of 18 January 2023. **a**, Continuously high concentrations of *N*_3–5_ particles are detected in an area with an extent of more than 300 km in two layers between 10.6–11.5 and 12.0–12.6 km. A cirrus cloud deck was present at altitudes below 10.8 km. **b**, High concentrations of dinitrates IP_2N_ were also detected. **c**, Vertical profile of the number concentration of *N*_3–5_ along the latitudinal cross-section, indicated by colour. **d**, Vertical profile of the number concentration of IP_2N_ along the latitudinal cross-section, indicated by colour. Satellite picture data in **a** and **b** obtained from https://wvs.earthdata.nasa.gov. Source Data

**Extended Data Fig. 9:**
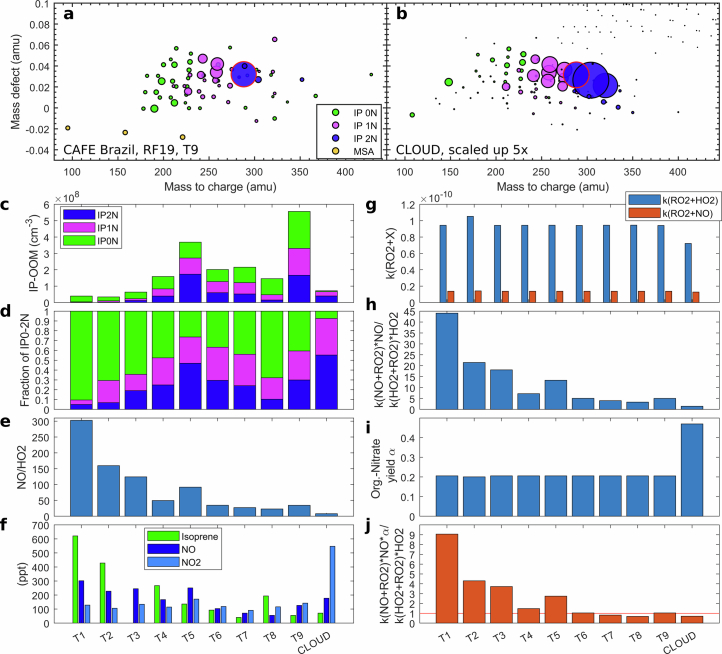
Comparison of isoprene-driven NPF of CAFE-Brazil RF 19 with the CLOUD isoprene + NO_*x*_ experiment. Mass-defect plots for NO_3_^−^-CIMS spectra from CAFE-Brazil, RF 19, T9, at −58 °C (same data as in Fig. 3a) (**a**) and Shen et al.^44^, adopted from their Fig. 3d, measured at the CLOUD chamber at −48 °C (**b**). Note that the original Fig. 3d in Shen et al. also contains the signals from NH_4_^+^-CIMS and Br^−^-CIMS instruments, which extends the range towards lower-oxygen-content molecules. Note also that their Fig. 3d shows the signals with the mass and mass defect of the reagent ion removed. We have added the effect of the NO_3_^−^ clustering here to make the data directly comparable with Fig. 3a of our study. Marker size is linearly scaled to concentration. The CLOUD NO_3_^−^-CIMS has a lower limit of detection (2 × 10^4^ cm^−3^ NO_3_^−^-CIMS at CLOUD versus 1 × 10^6^ cm^−3^ for the NO_3_^−^-CI-APi-TOF aircraft instrument at CAFE-Brazil) and thus can detect many smaller peaks. During RF 19, T9, the overall signal intensity of IP_0-2N_ was higher than in the CLOUD experiment, so the marker size in the lower panel has been scaled down by a factor of 5 for comparability. The red edge indicates C_5_H_10_O_2_(ONO_2_)_2_ clustered to a NO_3_^−^ reagent ion, which is the highest peak in RF 19, T9. Panels **c**–**j** show the conditions during time intervals T1–T9 from RF 19 (this study) and from the CLOUD isoprene + NO_*x*_ experiment^44^. **c**, Concentrations of IP_0N_, IP_1N_ and IP_2N_. **d**, Relative fractions of IP_0N_, IP_1N_ and IP_2N_. **e**, Ratio of NO to HO_2_. **f**, Mixing ratios of isoprene, NO and NO_2_. **g**, Rate coefficients for RO_2_ + HO_2_ and for RO_2_ + NO reactions. **h**, Rate coefficients *k*_RO2+HO2_ and *k*_RO2+NO_. **i**, Organonitrate yield *α*. **j**, Ratio of (*k*_NO+RO2_*[NO]*α)/(*k*_HO2+RO2_*[HO_2_]). Source Data

Our aircraft measurements, aggregated from 11 flights, show a pronounced diel cycle for the concentration of *N*_2–5_ particles, NPF events and isoprene oxidation products in the tropical upper troposphere (Fig. 4 and Extended Data Figs. 6 and 7). Here NPF events are defined conservatively from the differences of the 2-nm and 5-nm CN-counter channels by 0.7*N*_2_ − 1.3*N*_5_ > 0 (Methods). The concentration of *N*_2–5_ particles at night and in the early morning was generally small and no NPF events were detected before 08:00 local time. About 2 h after sunrise, we frequently detected high concentrations of *N*_2–5_ particles, dominating the total particle number concentration. Although *N*_2–5_ particle concentrations were negligible before the events, *N*_2–5_ was frequently greater than 10,000 scm^−^^3^ (converted to standard conditions according to the International Union of Pure and Applied Chemistry (IUPAC): 273.15 K and 1,000 hPa) during the events. NPF events were similar to those observed near the Earth’s surface in other environments in extratropical regions^34^, that is, in terms of abrupt occurrence of particles, strong initial growth, the role of photochemical production of the nucleating and condensable species and the large spatial extent of the events^34^. During a dedicated flight, the high *N*_2–5_ particle and organonitrate concentrations were observed in an area extending more than 300 km across and the two vertical layers in which the NPF was detected each extended over more than 0.5 km (Extended Data Fig. 8). During all flights, NPF events were associated with the occurrence of isoprene nitrates IP_1N_ and IP_2N_ (Fig. 4 and Extended Data Fig. 4). Shen et al.^44^ studied isoprene-driven NPF for upper-troposphere conditions around −30 and −50 °C with and without NO_*x*_ at the CLOUD chamber facility. For isoprene + NO_*x*_ conditions, both the gas-phase IP-OOMs spectrum as well as the resulting nucleation rates are in good agreement with our field study (see discussion in Methods, Extended Data Fig. 9 and Extended Data Table 1). Furthermore, Shen et al.^44^ show with APi-TOF measurements that isoprene organonitrates participate directly in the initial cluster formation. Although they found that, without NO_*x*_, IP-OOMs nucleate even stronger than in the presence of NO_*x*_, nitrate-dominated IP-OOMs still lead to substantial nucleation rates, which are in agreement with our findings. During CAFE-Brazil, for all observed NPF events, the CI-APi-TOF spectra were dominated by nitrate IP-OOMs, IP_1N_ and IP_2N_, as the presence of isoprene in the tropical upper troposphere is intrinsically coupled with high NO_*x*_ conditions through the NO_*x*_ production from lightning during the deep convective transport. We found strong NPF events during all five morning flights in the upper troposphere. Therefore, it seems likely that, at least at the beginning of the wet season (December to January), such events occur every morning over a large part of the Amazon, affected by the outflow of night-time deep convection accompanied by lightning.

The aggregated concentration measurements of the IP_0N_, IP_1N_ and IP_2N_ compounds show a similar diel cycle and altitude dependence as the *N*_2–5_ particles (Fig. 4). To be representative of pristine conditions, data that were measured during two encounters with biomass-burning plumes and data influenced by pollution emissions from the city of Manaus have been removed. Note that all data of Fig. 4 (as opposed to Figs. 1–3) were converted to standard temperature and pressure conditions for altitude-independent comparability and comparability with previous studies^1,2^. Overall, we observed NPF events during nearly 30% of measurements conducted at altitudes between 8 and 14 km and between 08:00 and 18:00 local time (Extended Data Fig. 7). At altitudes <8 km at any time and at >8 km during the night and in the early morning before 08:00 local time, NPF events represented less than 1%. Notable amounts of IP_0N_, IP_1N_ and IP_2N_ are also observed at altitudes <8 km, particularly in the boundary layer at midday, but the compounds are too volatile at these temperatures for NPF to occur (Figs. 3 and 4). We note that our measurements do not represent random averages, as the measurement flights had specific objectives, including the identification of NPF events in the upper troposphere.

## Atmospheric implications

The newly formed particles in the upper troposphere can be transported over large distances well beyond the Amazon region^27^. During transport, the aerosol size distribution changes owing to coagulation and continued condensation of low-volatile species. A fraction of the newly formed particles is expected to be transported downwards^38,40^, affecting the abundance of CCN at low altitudes in the tropics^1,6^. The modelling study in ref. ^4^ suggests that 35% of the low-altitude CCN were initially formed by nucleation in the free and upper troposphere^4^. Williamson et al.^1^ demonstrated that descending dry air in subsidence over the oceans contains more CCN-sized particles than moist air at low altitudes, indicating that NPF in tropical convective regions can increase the CCN concentrations of the lower troposphere. Because higher CCN concentrations can substantially influence the cloud microphysical and optical properties^8^, the process probably affects the atmospheric radiation budget and the climate^5^.

To put this into perspective, more than 7,000 mesoscale convective systems occur over the Amazon each year, covering a surface area of more than 40,000 km^2^ per system, typically lasting for 4 h (refs. ^53,54^) and with high lightning activity^21,55^. Therefore, NPF from the oxidation of biogenic isoprene combined with lightning NO_*x*_ may represent a notable mechanism of particle production in the tropical upper troposphere. It seems likely that this also occurs in the upper troposphere over other tropical forests, that is, in Central Africa, Southeast Asia and Northern Australia, that are also characterized by strong isoprene emissions^9,10^ and ubiquitous deep convection associated with lightning.

## Methods

The CAFE-Brazil mission took place in Brazil between 30 November 2022 and 29 January 2023, which covers the end of the dry-to-wet season transition and the beginning of the wet season in the Amazon. The German High Altitude and Long Range Research Aircraft (HALO) was stationed at Manaus International Airport, conducting a total of 16 local research flights (143 flight hours), as well as the transfer flights between Germany and Brazil (26 flight hours). An overview of the flight tracks is shown in Extended Data Fig. 1a. The research flights, at altitudes between 0.3 and 13.8 km, covered the region from 11° 33′ S to 4° 40′ N and from 72° 33′ W to 33° 50′ W. The aircraft was equipped with instrumentation to measure the in situ concentration of trace gases, radicals, aerosol number concentration and several aerosol physical and chemical properties. Descriptions of the methods used for the detection of the gas compounds and aerosol properties are given here.

### CI-APi-TOF mass spectrometer

The CI-APi-TOF mass spectrometer^56,57^ measures gaseous molecules that can contribute to NPF (that is, aerosol nucleation and initial growth up to detectable particle size). Gases such as isoprene oxidation products (IP_0,1,2N_), methanesulfonic acid (MSA) and sulfuric acid are detected. The instrument was specifically designed and certified for aircraft use and it is optimized for low inlet losses, a constant ionization pressure and minimal internal gas consumption.

The CI-API-TOF mass spectrometer uses a trace-gas inlet initially developed to measure OH radicals. It is almost identical to the inlet used for the HydrOxyl Radical measurement Unit based on fluorescence Spectroscopy (HORUS) instrument. The sampling location is placed at a sufficient distance from the fuselage to avoid the influence of the aircraft’s boundary layer. The inlet uses a set of shrouds and a flow restrictor to decelerate the air by a factor of 10 to around 25 m s^−1^ before sampling into the inlet line to reduce turbulence^58^. The flow restrictor, however, also causes a ram-pressure effect and thus adiabatic heating of the sample air depending on ambient pressure and air velocity, which will be discussed below in more detail.

The sampling line consists of a 20.5-mm inner diameter 1.8-m-long stainless-steel tube with an 8-mm orifice at the beginning (located in the centre part of the inlet). The sampling line has two bends with radii of about 120 and 500 mm, respectively, to enable installation close to the cabin wall. The inlet line is thermally insulated and has a temperature sensor 300 mm downstream of the sampling position inside the inlet. The sample flow is kept constant at 25.0 slpm for all altitudes to reduce wall losses, while still being in a laminar flow regime.

At the end of the sampling tube, the air reaches the SCORPION (Switchable Corona Powered Ion Source), which consists of a pressure-control and an ionization stage. The pressure-control stage comprises two sequentially placed and conically shaped orifices with 1.4 mm inner diameter each. Between these two orifices, a PID-controlled solenoid regulation valve allows for variable pumping, which provides a constant pressure of 200 hPa in the ionization region. The ionization region is located directly after the second orifice. It consists of a 20.5-mm inner diameter stainless-steel tube and two orthogonally attached ion source units, of which only one is used at any given time. The ion source uses a corona discharge to produce (HNO_3_)_0,1,2_NO_3_^−^ reagent ions from gaseous HNO_3_. Because a corona discharge also produces OH radicals, which could alter the chemical composition of the sample air, we implemented a counter-flow regime, such that the nitrate reagent ions are pushed towards the sample air by means of an electric field, whereas the gas flow, which also carries OH radicals, is directed away from the sample air towards the exhaust.

The nitrate reagent ions are mixed with the sample air and travel along the main drift tube (130 mm length) with a reaction time between 180 and 350 ms, depending on altitude. Although the ionization pressure is kept constant at 200 hPa, the flow along the drift region varies between 2.7 slpm at ground level and 1.4 slpm at 12 km altitude, causing different reaction times. The higher flow at lower altitudes is needed to maintain the ionization pressure at 200 hPa with the given orifice diameters. Although the reaction times are around 3–5 times longer than in an Eisele–Tanner-type ion source^59^ (about 50 ms), the ionization pressure is also lower by a factor of 5, leading to a roughly comparable number of collisions between reagent ions and sample gas. However, because of the extra orifices in the pressure stage and a relatively long inlet line, the overall sensitivity of SCORPION is lower by about one order of magnitude compared with a nitrate reagent ion long time-of-flight (LTOF) mass spectrometer instrument as deployed, for example, at the CLOUD chamber^42,43^. The detection limit for SCORPION is between 5 × 10^5^ and 5 × 10^6^ cm^−3^.

At the end of the SCORPION drift tube, the ions enter the Tofwerk time-of-flight mass spectrometer by means of a 350-µm inner diameter orifice. We use an HTOF with a resolution of around 4,000 at the *m*/*z* range 250–300 amu. The data are recorded at 1 Hz; however, it is averaged to 10 s resolution before post-processing and high-resolution peak fitting in Tofware (version 3.2.5, Aerodyne).

To derive the ambient concentrations of IP_0,1,2N_, several pressure-dependent and temperature-dependent correction factors have to be applied to the fitted peak intensities as follows:

$$\begin{array}{l}[{{\rm{IP}}}_{{\rm{0,1,2N}}}]=A({p}_{{\rm{a}}},{p}_{{\rm{i}}}{,T}_{{\rm{a}}}{,T}_{{\rm{i}}})\times I({p}_{{\rm{i}}},{T}_{{\rm{i}}},{F}_{{\rm{i}}})\\ \,\,\,\,\times \left(C({p}_{{\rm{i}}})\times {\rm{ln}}\left(1+\frac{{({{\rm{IP}}}_{{\rm{0,1,2N}}})}_{{\rm{cps}}}}{{\rm{Tr}}\left(\frac{m}{z}\right)\sum {{{\rm{NO}}}_{3}}^{-}{({{\rm{HNO}}}_{3})}_{i=0,1,2}}\right)-{\rm{BG}}\right)\end{array}$$

Here (IP_0,1,2N_)_cps_ represents the fitted peak intensities in counts per second. Tr(*m*/*z*) is the *m*/*z*-dependent correction factor for the relative change in instrument transmission efficiency^60^. For the IP_0,1,2N_ *m*/*z* range, this correction is about 10–20%, depending on the exact *m*/*z* ratio.

A pressure-dependent calibration factor is applied to the normalized signal (at 12.2 km altitude, *C*_230hPa_ = 6.5 × 10^10^ cm^−3^, whereas at ground level, *C*_1000hPa_ = 2.1 × 10^11^ cm^−3^). This calibration factor was experimentally estimated for gaseous sulfuric acid by generating a known amount of gaseous sulfuric acid by means of ultraviolet (UV)-induced OH production (from photolysis of H_2_O) and subsequent oxidation of sulfur dioxide^61^ to sulfuric acid^62^. We constructed a dedicated calibration rack for the CI-APi-TOF mass spectrometer, similar to that described in ref. ^62^. The calibration unit can be operated at pressures between 200 and 1,000 hPa.

After applying the calibration factor, a background correction of the signal is performed (BG). Background measurements were performed in flight by overflowing SCORPION with synthetic air from the internal gas bottle. This can, however, only be done above 9 km altitude, as at lower altitudes more gas would be needed to overflow the ion source than provided by the internal gas bottle. Typically, 2–3 background measurements are performed per flight, each lasting 10 min.

The factor *I*(*p*_i_, *T*_i_, *F*_i_) represents the correction for losses to the wall in the 1.8-m-long sampling line and depends on inlet pressure *p*_i_, inlet temperature *T*_i_ and inlet flow *F*_i_. It is based on the parametrization for straight tube losses and thus ignores the two curves in our inlet tube. The inlet loss estimation uses the experimentally determined diffusion coefficient for gaseous sulfuric acid and its pressure-dependent and temperature-dependent parametrizations^61^. The inlet correction factor is about 1.65 for a typical high-altitude flight scenario (12 km altitude).

The correction factor *A*(*p*_a_, *p*_i_, *T*_a_, *T*_i_) scales the concentration levels from inlet temperature *T*_i_ and pressure *p*_i_ conditions to ambient temperature *T*_a_ and pressure *p*_a_ conditions. This scaling is necessary as the ram pressure caused by the flow restrictor increases the inlet pressure compared with ambient conditions (at 12.2 km altitude, at which most of research flight (RF) 19 was flown from 188 hPa ambient pressure to 268 hPa inlet pressure). This pressure increase also induces an adiabatic heating of the sampling air. Further heating comes from the limited thermal insulation of the inlet line. At 12.2 km altitude, we measure a temperature increase from −58 °C (ambient) to −13 °C (inlet), so Δ*T* = 45 °C. Although the inlet residence time at these conditions is rather short (0.38 s), this temperature increase could lead to the evaporation of molecules from the aerosol to the gas phase, which could enhance our measured gas-phase signals. At −58 °C, the main IP_0,1,2N_ products are ELVOCs, whereas they shift to the low-volatility organic compounds range at −13 °C (Fig. 3). Especially for freshly nucleated particles with diameters in the size range in which the Kelvin effect has a role, this could cause evaporation. Therefore, the measurements are considered to be a combination of pure gas-phase concentration and potentially re-evaporated aerosol-phase molecules. However, even if a large fraction of our signal would originate from evaporation of freshly nucleated particles, this still highlights the crucial role of isoprene-derived oxidation products for the NPF process in the upper troposphere over the Amazon.

The measurement of sulfuric acid was affected by a sulfur contamination inside the stainless-steel vessel containing the liquid HNO_3_ supply of the ion source. This led to an increased instrumental background for sulfuric acid of about 2 × 10^6^ cm^−3^ for our measurements at altitudes above 8 km.

The overall uncertainty of the CI-APi-TOF measurements is ±62%. This consists mainly of the uncertainty of the sulfuric acid calibration factor of 55%, as well as the uncertainty of the mass-dependent transmission correction (20%) and the inlet loss correction (20%). No calibration standards are available for the measurement of ELVOCs from isoprene or monoterpenes. Therefore, we use the same calibration factor as determined for the sulfuric acid measurements. The sensitivity of the CI-APi-TOF instrument is high for the detection of highly oxidized organics with many functional groups, for example, HOMs from monoterpenes. For those, this approach is well established^42^, but the CI-APi-TOF instrument is expected to be less sensitive for the smaller IP-OOMs, especially when the molecules contain few oxygen atoms. This general dependence is confirmed by the comparison of nitrate reagent ions with, for example, bromide or iodide reagent ions^41^. The concentrations of the isoprene oxidation products detected by the CI-APi-TOF mass spectrometer should therefore be considered as lower limits. Compared with the IP_0N_, IP_1N_ and IP_2N_ reported in ref. ^44^, the CI-APi-TOF instrument will detect smaller fractions of these compound classes, as more mass spectrometers using other reagent ions are used in ref. ^44^ to complement the IP_0,1,2N_ measurements. Nevertheless, the relative changes of individual compounds over time, as shown, for example, in Extended Data Fig. 5, are not affected by this effect.

We also apply the calibration factor estimated for sulfuric acid to derive the concentration of IP_0-2N_, as a direct calibration for IP_0-2N_ is not possible. It was shown that HOMs with sufficient oxygen content, that is, a sufficient number of functional groups to cluster with NO_3_^−^, are charged with nitrate reagent ions at the kinetic limit, similar to sulfuric acid^41,63^. Riva et al.^41^ conducted a detailed comparison of various reagent ions and their respective sensitivities towards pure organic as well as nitrate HOMs derived from monoterpene oxidation. They found that both nitrate and non-nitrate HOMs can be charged by NO_3_^−^ reagent ions at the kinetic limit as long as HOMs contain more than six oxygen atoms in the non-nitrate case and more than seven oxygen atoms in the nitrate case. Assuming that these results are transferable to IP_0-2N_, this means that some of our reported IP_0-2N_ could be charged below the unit charging efficiency of the kinetic limit. This leads to an increase in calibration factor for these species and our reported concentrations represent a lower limit estimation, especially for low-oxygen-content IP_0-2N_.

### Aerosol number concentration and nucleation-mode particles

The Fast Aerosol Size Distribution (FASD) instrument is a compact multichannel system to detect newly formed particles. It was developed specifically for operation aboard the HALO aircraft. The system combines the concept of well-established commercial ultrafine condensation particle counters^64^ (CPCs) with central temperature management, central butanol vapour supply, central pressure control and a new type of flow system. The prototype, designed and built at the Max Planck Institute for Chemistry (MPIC), simultaneously measures aerosol concentration in ten channels every second. The ten-channel device is about the size of two commercial ultrafine CPCs. Similar aircraft-based instruments use several CPCs^65–67^.

For each CPC channel, the sample air is fed by means of a short (6 mm) capillary into a heated mixing chamber, surrounded by particle-free butanol-rich sheath air and fed into the cold condensation region. The supersaturated butanol condenses on the aerosol particles, causing them to grow sufficiently to be detected in a downstream optical particle counter. To minimize diffusional losses, all channels share a central sample line with a distance of only about 38 mm between adjacent inlets and the inlet capillaries protrude into the core sample flow.

The FASD instrument gradually reduces the butanol content of each sheath flow in a dilution chamber between the saturator and the channels. The exhaust air from each channel is collected and cleaned of particles by a HEPA filter and returned to the saturator and dilution stage by a central pump. There is a total of four pressure stages in the sheath flow loop, with the highest pressure in the saturator and the butanol dry air upstream of the dilution. Two valves control the saturated and dry air flow ratio into the dilution chamber. Although the saturated air is introduced at a single point, the dry air flow is split to replenish the air flowing to each channel. The mixing, condensation and detection areas of each channel are close to the ambient pressure level in the inlet and outlet lines. Finally, the lowest pressure is upstream of the sheath flow pump and also drives the removal of butanol that condenses on the walls of each condenser. This butanol is collected in a reservoir and can be reused as normally no water vapour condenses.

A valve controls the total sample flow. This valve allows some air to leave the sheath flow loop from the dilution stage to the exhaust line. In total, an equal amount of air enters through the short inlet capillary of each channel. Another valve sets the desired flow through the dilution stage and allows some air to flow directly from the end of the dilution stage to the sheath flow pump. Flow uniformity is achieved by carefully selecting the flow resistances, which results in pressure differentials between stages in the range 10–20 hPa. The condenser sections are cooled to a target temperature of 10 °C by a water cooling system. The waste heat is transferred from the cold water cycle to the hot water cycle through Peltier elements, in which the heat is eventually released to the ambient air through an external radiator. To avoid unwanted butanol condensation, it is essential that the dilution chamber and the individual sheath flow transfer lines and mixing chambers of each channel maintain temperatures above the saturator temperature. This is achieved by heating the saturator indirectly, while keeping the other parts actively warm, resulting in a permanent temperature gradient that prevents unwanted condensation.

The particle activation is determined by the temperature difference between condensation and saturation, the pressure differentials and the dilution factor. By measuring the activation curves under given conditions and comparing them with theoretical calculations, the activation behaviour can be derived for fluctuating measurement conditions^65^. Initial measurements of selected particle sizes in the range 2–30 nm show that the performance of the first channel of the prototype is comparable with a commercial ultrafine CPC, whereas the measured values of the other channels fit to an effective dilution to around 75% per channel.

Although diffusional losses between the channels are not apparent, the final calibration requires further measurements^65,67^. Therefore, for this study, the theoretical cut-off diameters are obtained using Kelvin’s equation^68^ with a dilution of 75% per stage. For each measurement time step, the corresponding particle activation diameters can be calculated from the actual temperatures and pressure differences and are typically in the range 2–6 nm. Measurement periods without NPF events can be used to determine systematic deviations of sample flows owing to slight variations in flow resistances. To keep the measurement conditions of the channels stable, regardless of changes in ambient pressure, the FASD instrument is operated at a constant pressure of typically 200 hPa. The pressure control is achieved by continuously calculating the net flows in and out of the FASD instrument based on flow controller and pressure sensor data. The calculation results are used to drive two PID controllers that regulate the outflow by using a mass flow controller and the inflow by using a custom-made size-changing orifice^69^.

### Proton transfer reaction time-of-flight mass spectrometry

A proton transfer reaction time-of-flight mass spectrometer^46,70,71^ (PTR-TOF-MS 8000, Ionicon Analytik) was used for the fast high-mass-resolution airborne measurements of VOCs (*m*/*z* < 500 amu). Isoprene, its oxidation products including methyl vinyl ketone, methacrolein and isoprene hydroxyhydroperoxide, and total monoterpenes reported in this study were measured at *m*/*z* = 69.069, 71.049 and 137.132 amu, respectively. In this technique, hydronium ions (H_3_O^+^) are used as a reagent to ionize molecules in air that have a higher proton affinity than water (693 kJ mol^−1^). The instrument was operated with a drift pressure of 2.2 mbar and an *E*/*N* of 137 Td. Air was drawn from outside the aircraft to the instrument through a fuselage-mounted inlet housing, in a heated 2-m-long, 0.64-cm outer diameter Teflon inlet line. Quantification of the detected compounds was performed by frequent in-flight background determinations with zero air and several ground-based calibrations using a gravimetrically prepared gas standard containing isoprene, methyl vinyl ketone and α-pinene (Apel-Riemer Environmental). At a time resolution of 1 min, the detection limit (3*σ*) was calculated to be 100, 41 and 25 pptv for isoprene, its oxidation products and total monoterpenes, respectively. An ozone correction for isoprene was applied on the basis of laboratory experiments and comparison with the gas chromatography–mass spectrometry data. The total uncertainty of measurement was usually below 25%. Further detailed information about the proton transfer reaction time-of-flight mass spectrometry used is given ref. ^72^, its response to atmospheric ozone in ref. ^73^ and the configuration in the aircraft is described in ref. ^74^.

### Gas chromatography–mass spectrometry

VOCs were measured in situ using a customized gas chromatograph coupled to a commercial quadrupole mass spectrometer (Agilent Technologies 5973 MSD). The system has been described in detail previously^75^ and its configuration in the aircraft payload for the CAFE-Brazil campaign is given elsewhere^74^. In brief, ambient air is drawn at 200 scm^3^ through the Trace Gas Inlet (TGI, Enviscope) to the instrument by means of a heated 2-m-long Teflon line (0.6 cm outer diameter) equipped with a sodium thiosulfate ozone scrubber^73^. Within a series of traps in a liquid-nitrogen-cooled cryo-concentrator, the sampled air is dried (−10 °C), enriched for VOCs (−160 °C) for 1 min and then concentrated into a small volume (−160 °C) before being rapidly heated to inject the sample into the gas chromatograph. The compounds are separated by a DB-624 UI, 10 m, 0.25 mm, 1.4 µm capillary column (Agilent Technologies). The temperature programme of the custom-built gas chromatograph oven is as follows: 30 °C for 50 s, then 30 °C to 200 °C at 1.8 °C s^−1^ and constant 200 °C for the rest of the chromatogram. After separation, the compounds are electronically ionized (70 eV) and detected by the mass spectrometer in the selected ion mode. In the configuration used for the CAFE-Brazil campaign, more than 35 compounds could be resolved and quantified in a 2.4-min chromatogram, the overall measurement frequency being 3 min. Calibration was achieved using a gravimetrically prepared multicomponent pressurized standard (Apel-Riemer Environmental), with a stated accuracy of 5%, with calibrations being performed before, during and after each flight. Isoprene was detected at *m*/*z* = 67 amu and a retention time of 0.7 min with a detection limit of 5 pptv and an uncertainty of about 10%.

### NO_*x*_ measurements

Nitrogen oxides were measured through photolysis chemiluminescence with the two-channel instrument Nitrogen Oxides Analyzer for HALO (NOAH). In one channel, nitric oxide (NO) is converted to excited-state nitrogen dioxide (NO_2_*) by reaction with excess amounts of ozone (O_3_). A photon is emitted during de-excitation of NO_2_*, which is detected by a photomultiplier tube. The signal is converted to ambient NO mixing ratios using normal on-ground calibrations between the flights. The second channel is identical except for using a photolytic converter, in which NO_2_ is photolysed to NO at a wavelength of around 395 nm before the addition of O_3_. The conversion efficiency of the converter (the fractional conversion of NO_2_ to NO) was 29% during the CAFE-Brazil campaign. Owing to enhanced temperatures in the instrument and the photolytic converter, NO_2_ reservoir species, mostly methyl peroxy nitrate, can release NO_2_. Therefore, the NO_2_ measurement represents a sum of NO_2_ and thermally labile nitrates and represents an upper limit of the NO_2_ mixing ratios at night-time. For the day-time data, we use NO_2_ derived from the photostationary state (inferred from NO, O_3_ and *j*(NO_2_)) in this study instead (Extended Data Fig. 2). The 1 Hz detection limit for NO is 8 pptv and the overall measurement uncertainty is 5%. A detailed description of the instrument is presented in refs. ^76^,^77^. Details on the thermal decomposition of NO_2_ reservoir species in photolytic converters and resulting interferences can be found, for example, in refs. ^77,78,79^.

### HO_*x*_ measurements

The airborne HORUS instrument is based on the Fluorescence Assay by Gas Expansion–Laser-Induced Fluorescence of OH (FAGE-LIF) instrument as described in detail in ref. ^80^. It was developed specifically for operation on the HALO research aircraft and combines an external inlet shroud with an in-flight calibration system, OH and HO_2_ detection axes, a laser system and a vacuum system. The OH is drawn into the detection axis through a critical orifice at a pressure range of 300–1,300 Pa, depending on ambient pressure. It is selectively excited on the *Q*1(2) transition line (A2Σ +−X2Π, ν′ = 0, ν″ = 0) by a 3-kHz pulsed UV laser light around 308 nm. The UV laser emission wavelength is periodically tuned on and off resonance of the OH *Q*1(2) transition to quantify the fluorescence background. An inlet pre-injector (IPI) system is installed to remove atmospheric OH to measure the chemical OH background signal. The airborne IPI system has been redesigned to fit within the inlet shroud system, while maintaining similar operational features as the on-ground IPI installation^81^.

HO_2_ is measured indirectly through the quantitative conversion of atmospheric HO_2_ to OH by injection of NO within HORUS.

1 $${{\rm{HO}}}_{2}+{\rm{NO}}\to {{\rm{NO}}}_{2}+{\rm{OH}}$$

With excess amounts of NO used in the conversion of HO_2_ to OH, subsequent HONO formation has to be taken into account.

2 $${\rm{OH}}+{\rm{NO}}+{\rm{M}}\to {\rm{HONO}}+{\rm{M}}$$

The losses resulting from internal HONO formation are dependent on pressure and amount to less than 1% above 10 km and less than 2% at ground level.

We account for the reaction of RO_2_ with NO leading to HO_2_ formation, which then generates OH in the presence of NO. We reduce the NO addition to limit the contribution of RO_2_ to the detected OH levels. The instrument operates in two modes during flights: one with a low NO addition, creating an internal NO concentration of 1.5 ± 0.1 × 10^13^ cm^−3^ and achieving a conversion rate of 20–40% depending on altitude, and another with a high NO addition of 7 ± 0.1 × 10^13^ cm^−3^, reaching a conversion rate of more than 95%. Furthermore, NO titrations are conducted at different altitudes to ensure accurate measurement of the HO_2_ contribution. The HORUS instrument 1*σ* accuracy is ±22.6% for OH and ±22.1% for HO_2_. Precision depends on ambient pressure and instrument performance.

### Aerosol mass spectrometer

The composition of non-refractory aerosol particles in the diameter range approximately 50–800 nm was measured using a compact time-of-flight aerosol mass spectrometer (C-ToF-AMS)^82,83^. The instrument samples the aerosol particles by means of a constant pressure inlet and an aerodynamic lens into the vacuum system. The particles are flash vaporized on a 600 °C surface and the resultant gas-phase molecules are ionized by electron ionization. The ions are analysed by a time-of-flight mass spectrometer. The C-ToF-AMS has been operated on HALO since the ACRIDICON-CHUVA campaign in 2014, which also took place over the Amazon rainforest^31^.

### Other measurements

Carbon monoxide was measured with the quantum cascade laser absorption spectrometer TRISTAR with a mean total measurement uncertainty of 3.5% (refs. ^84,85^).

Upward and downward spectral actinic flux densities in the range 280–650 nm are measured by combinations of two CCD spectroradiometers^86,87^.

The Fast AIRborne Ozone (FAIRO) instrument measures ozone with high temporal resolution (10 Hz). It combines two independent techniques, UV photometry and chemiluminescence detection^88^.

Data from the Basic HALO Measurement and Sensor System (BAHAMAS) are used for determination of the aircraft position, wind velocity and direction, humidity, temperature and pressure^89^.

### Trajectory calculations

Quasi-Lagrangian sampling periods during HALO flights were identified in Fig. 1 (marked by grey shading) using the flight measurements in combination with backward trajectories as described in the following.

The sampling period with the highest number of *N*_2–5_ particles (light-red shading in Fig. 1 (T9)) was chosen as the reference period, with its limits defined as 10:05:46 and 10:10:30 local time. During this sampling period, HALO covered a distance of 62.2 km, which becomes relevant during the final computational step determining the quasi-Lagrangian sampling periods. To identify time intervals earlier during the flight in which approximately the same air mass as during the reference period was examined, backward trajectories were calculated for a set of ten parcels. These were initialized at HALO’s instantaneous location every 30 s (at 0 s and 30 s times) during the reference period. We used a simple Euler scheme with a 30-s time step to calculate the trajectories. We further assumed a constant wind velocity for each trajectory. The wind speed and direction were based on HALO measurements at the parcel initialization time. Vertical movements were not considered. These computations result in an *m* × 10 matrix containing the ten position vectors of the parcels initialized at HALO’s location during the reference period (*p*_1_, *p*_2_,…, *p*_10_) at each historical time (*t*_*i*_, *t*_*i*_ − Δ*t*,…, *t*_*i*_ − (*m* − 1)Δ*t*). We then calculated the Euclidean distance from each parcel to HALO’s location (in km), at each location along the trajectories. This distance is always computed with respect to HALO’s instantaneous position at the equivalent time. Finally, the quasi-Lagrangian intervals highlighted by the grey shading in Fig. 1 (T1–T8) were determined by selecting time periods along the backward trajectories for which the mean distance from the parcels to HALO was less than the sampling distance of the reference period (that is, 62.2 km).

The trajectories shown in Fig. 2 were computed using the above described method, but instead initializing 1-min-spaced parcels between 08:05 and 08:15 local time.

It should be noted that the calculated trajectories involve uncertainties, mainly because of the assumption of constant wind speed and the neglect of vertical velocity. However, tests, in which further backward trajectories were computed from each semi-Lagrangian time interval itself (not shown), indicate that the time-dependent changes in wind speed from one phase of the flight pattern loop to another did not substantially affect the estimates of the air-mass location. This indicates that we may use the approximation of constant horizontal wind speed on the spatio-temporal scales analysed for this specific flight. Also, convective clouds typically induce atmospheric gravity waves in their surroundings. Consequently, air parcels expelled by convective outflows may oscillate in the vertical dimension during horizontal displacements, remaining close to the outflow level for a few hours. Thus, vertical errors in parcel location are expected to be within a range smaller than the spatial extent of the air mass in which NPF was identified (about 62 km). We thus argue that neglecting the vertical velocity component serves as a reasonable proxy for the air-mass locations before the measurements in the near outflow proximity, in the absence of sufficiently accurate time-dependent vertical wind-speed data. This assumption also immediately breaks down when the backward trajectories come in contact with active convection.

Given the absence of a true value to evaluate parcel trajectories at this scale (note that atmospheric models provide time-dependent three-dimensional wind velocities, but these are highly sensitive to the representation of the location and structure of convective storms, making them unsuitable for this type of high-resolution analysis), the final validation should come from the tracer measurements themselves. The strong agreement between this trajectory analysis and the in situ measurements is reassuring and provides some validation of the conclusions presented here. Note that, during T8, only small amounts of *N*_2–5_ particles and IP_0-2N_ were found, indicating that, at this time, the centre of the NPF air mass had probably moved north of section BC of the flight track (Fig. 2).

### Identification of NPF

To identify a NPF event, we conservatively assumed a measurement uncertainty of 30% for each channel of the FASD instrument (including statistical uncertainty, drifts in the flows and other systematic uncertainties). The difference between *N*_2_ and *N*_5_ is classified as a NPF event if 0.7*N*_2_ − 1.3*N*_5_ > 0 cm^−3^ (refs. ^28,66^).

### Condensation sink

The condensation of a vapour to aerosol particles is described by the CS (ref. ^90^). For vapours of ultralow or extremely low volatility, the condensation to pre-existing large particles competes with the NPF process. The size distribution of the aerosol is crucial for determining the CS. An Ultra-High Sensitivity Aerosol Spectrometer (UHSAS) was generally used in combination with the FASD CPC measurements to determine the aerosol size distribution in the size range 60–1,000 nm, but for RF 19, the UHSAS was not operational. Therefore, we performed a rough estimation of the range of the upper limit of the CS by assuming that all aerosol particles measured by the 5-nm FASD channel *N*_5_ have a size of 20, 50 or 100 nm. These three estimates are given in Extended Data Figs. 2 and 3. Before and outside the NPF events, the 50-nm or 100-nm assumptions give a reasonable range (compared with the other research flights when the UHSAS was operational), whereas during the NPF events, the <20-nm assumption is more likely to be correct.

### Saturation concentration

The SIMPOL model is used to obtain an estimate of the temperature-dependent saturation concentration *C*_i_* for the isoprene oxidation products^49^ (Fig. 3). SIMPOL is based on the group-contribution method, in which the number of functional groups of an organic molecule determines its saturation concentration. For the SIMPOL-derived saturation concentration, an uncertainty of one order of magnitude in the volatility distribution is assumed^43^. The saturation ratio *S*_i_* of a compound can be determined by calculating *S*_i_* = [*c*_*i*_]*m*_*i*_/(*N*_A_*C*_i_*), with [*c*_*i*_] denoting the concentration of compound *c*_*i*_ and *m*_*i*_ its molecular mass. The saturation ratio, *S*_i_*, of a compound can then be compared with estimates of the Kelvin diameters beyond which condensation is favoured over evaporation (Extended Data Fig. 3).

### Comparison of CAFE-Brazil results to the CLOUD laboratory measurements of Shen et al.

Shen et al.^44^ report laboratory measurements from the CLOUD chamber that investigate the role of isoprene for NPF at cold upper troposphere conditions (around −30 and −50 °C) with and without NO_*x*_. For isoprene + NO_*x*_ conditions at −48 °C, they report an IP-OOMs distribution (Fig. 3d in ref. ^44^) that is similar to the one we report for our atmospheric measurements (Fig. 3a and Extended Data Fig. 9). Note that the figure in ref. ^44^ shows not only IP-OOMS data from a NO_3_^−^ reagent ion CIMS similar to the CI-APi-TOF used in our study, but, in addition, data from IP-OOMs measured by further mass spectrometers using NH_4_^+^ and Br^−^ reagent ions. This extends the range of detected compounds towards higher volatility compounds with lower oxygen content^41,44^. These extra compounds are not expected to drive nucleation on their own but may contribute to the growth of newly formed particles after they have reached the respective Kelvin diameter. Comparing only the NO_3_^−^ reagent ion data, the CLOUD data match our mass-defect plot (Extended Data Fig. 9). Nitrates, especially dinitrates, dominate the range in both cases. In ref. ^44^, the NO_3_^−^-CIMS measurement shows an even stronger dominance of nitrate IP-OOMs than our study. The highest peak in our study (C_5_H_10_N_2_O_8_) is the second highest peak in ref. ^44^, whereas the highest peak found by Shen et al. is the closely related C_5_H_10_N_2_O_9_, which can be formed by low-temperature RO_2_ reaction with NO_2_ instead of NO (ref. ^44^). This is more likely in CLOUD as it was operating at a higher NO_2_/NO ratio owing to lower NO_2_ photolysis rates in CLOUD compared with the upper troposphere over the Amazon during daytime (CLOUD: NO_2_/NO ≈ 3.1; CAFE-Brazil, RF 19, T9, NO_2_/NO ≈ 1.1; Extended Data Fig. 9 and Extended Data Table 1). Nevertheless, there is good overall agreement between the spectra recorded in CLOUD and our study.

Shen et al.^44^ report that non-nitrate IP-OOMs are more effective for nucleation than nitrate IP-OOMs, which can be seen from lower nucleation rates at comparable concentration (extended data figure 5 in ref. ^44^). Even with the lower nucleation efficiency for isoprene nitrates, the authors still measured notable nucleation rates at −48 °C in a nitrate-IP-OOMs-dominated experiment (*J*_1.7_ ≈ 4 cm^−3^ s^−1^ for 2 × 10^8^ cm^−3^ of IP_1-2N_) with a gas-phase IP-OOMs spectrum similar to the one we report, as discussed above. Furthermore, figure 3c in ref. ^44^ confirms that nitrate-IP-OOMs do participate in initial cluster formation. The authors also state that the role of nitrates for nucleation could increase at colder temperatures, as encountered during our flights (−58 °C for RF 19). They report a more than 100-fold increase in nucleation rate for isoprene + NO_*x*_ conditions when the temperature is reduced from about −30 to −50 °C. Given the observation that, at −50 °C, nitrate-IP-OOMs are weaker nucleators than non-nitrate-IP-OOMs, which means that they do not nucleate at the kinetic limit at −50 °C, it is plausible that the nitrate-IP-OOMs-driven nucleation rate increases when the temperature is reduced from −50 °C to −58 °C. Although the CLOUD experiment could only measure as cold as −50 °C, the expected increase in nucleation rate at colder temperatures leads to a good agreement with the NPF rate of 20 cm^−3^ s^−1^ for a concentration of 2.7 × 10^8^ cm^−3^ of IP_1-2N_ reported here. We note that, even if nitrates dominate the IP-OOMs spectra, we do not rule out an important contribution of non-nitrates to the initial steps of cluster formation and nucleation.

Overall, the results of the Shen et al.^44^ laboratory study agree with the nitrate-dominated IP-OOMs spectra that were measured in the upper troposphere over the Amazon. Taking into account the lower temperatures during our flights, the formation rates measured in the laboratory for isoprene + NO_*x*_ conditions are comparable with those estimated in our study.

The probable reason for why nitrate IP-OOMs are much more prevalent than non-nitrate-IP-OOMs is that the presence of isoprene and NO_*x*_ in the upper troposphere over the Amazon region is intrinsically coupled through deep convection, as isoprene is transported rapidly from the boundary layer to the upper troposphere, and NO_*x*_ is produced by lightning. Both isoprene and NO_*x*_ accumulate during the night. After sunrise, photolysis leads to the production of OH and NO (with NO presence enhancing OH recycling as well). OH and NO then trigger isoprene oxidation and the corresponding RO_2_ termination reactions that lead to the reported nitrate-dominated IP-OOMs spectra and NPF events.

## Online content

Any methods, additional references, Nature Portfolio reporting summaries, source data, extended data, supplementary information, acknowledgements, peer review information; details of author contributions and competing interests; and statements of data and code availability are available at 10.1038/s41586-024-08192-4.

## Source data

Source Data Fig. 1

Source Data Fig. 2

Source Data Fig. 3

Source Data Fig. 4

Source Data Extended Data Fig. 1

Source Data Extended Data Fig. 2

Source Data Extended Data Fig. 3

Source Data Extended Data Fig. 4

Source Data Extended Data Fig. 5

Source Data Extended Data Fig. 6

Source Data Extended Data Fig. 7

Source Data Extended Data Fig. 8

Source Data Extended Data Fig. 9

Source Data Extended Data Table 1

## Figures and Tables

**Extended Data Table 1:**
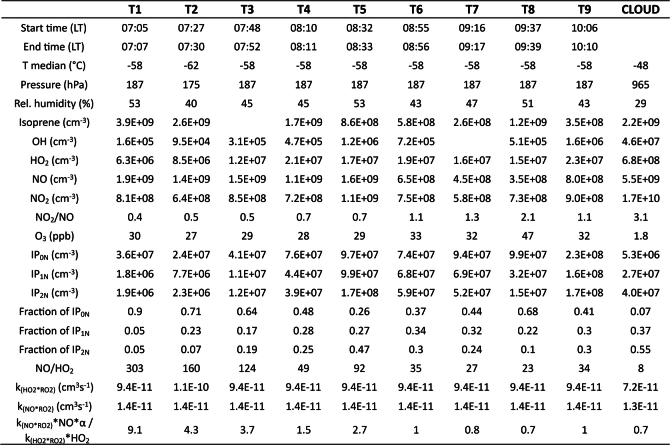
Comparison of NPF conditions during CAFE-Brazil RF 19 with the CLOUD experiment Source Data

Median concentrations, rate constants and meteorological parameters for each time interval (T1–T9) of RF 19, as well as data from Shen et al.^44^, for an isoprene + NO_*x*_ experiment at the CLOUD chamber (same experiment as shown in Fig. 3d in ref. ^44^). For retrieving the values within each T interval, we selected a time frame corresponding to measurements from a consistent air mass, as determined by the distinct enhancements in our experimental data. Note that, for determining IP_0,1,2N_ for CLOUD Fig. 3d (last column) and their fractions, only data from the NO_3_^−^-CI-APi-TOF is used.

## Data Availability

The full dataset shown in the figures is publicly available at 10.5281/zenodo.12527358 (ref. ^91^). Source data are provided with this paper.

## Code availability

Data-analysis routines used to process data and generate plots are available from the corresponding author on reasonable request.
