# Author Correction: Isoprene nitrates drive new particle formation in Amazon’s upper troposphere

**DOI:** 10.1038/s41586-025-08906-2

**Published:** 2025-04-01

**Authors:** Joachim Curtius, Martin Heinritzi, Lisa J. Beck, Mira L. Pöhlker, Nidhi Tripathi, Bianca E. Krumm, Philip Holzbeck, Clara M. Nussbaumer, Lianet Hernández Pardo, Thomas Klimach, Konstantinos Barmpounis, Simone T. Andersen, Roman Bardakov, Birger Bohn, Micael A. Cecchini, Jean-Pierre Chaboureau, Thibaut Dauhut, Dirk Dienhart, Raphael Dörich, Achim Edtbauer, Andreas Giez, Antonia Hartmann, Bruna A. Holanda, Philipp Joppe, Katharina Kaiser, Timo Keber, Hannah Klebach, Ovid O. Krüger, Andreas Kürten, Christian Mallaun, Daniel Marno, Monica Martinez, Carolina Monteiro, Carolina Nelson, Linda Ort, Subha S. Raj, Sarah Richter, Akima Ringsdorf, Fabio Rocha, Mario Simon, Sreedev Sreekumar, Anywhere Tsokankunku, Gabriela R. Unfer, Isabella D. Valenti, Nijing Wang, Andreas Zahn, Marcel Zauner-Wieczorek, Rachel I. Albrecht, Meinrat O. Andreae, Paulo Artaxo, John N. Crowley, Horst Fischer, Hartwig Harder, Dirceu L. Herdies, Luiz A. T. Machado, Christopher Pöhlker, Ulrich Pöschl, Anna Possner, Andrea Pozzer, Johannes Schneider, Jonathan Williams, Jos Lelieveld

**Affiliations:** 1 Institute for Atmospheric and Environmental Sciences, Goethe University Frankfurt, Frankfurt am Main, Germany; 2 Atmospheric Microphysics Department, Leibniz Institute for Tropospheric Research, Leipzig, Germany; 3 Faculty of Physics and Earth Sciences, Leipzig Institute for Meteorology, Leipzig University, Leipzig, Germany; 4 Max Planck Institute for Chemistry, Mainz, Germany; 5 Lemon Labs Ltd., Nicosia, Cyprus; 6 Department of Environmental Science, Stockholm University, Stockholm, Sweden; 7 Bolin Centre for Climate Research, Stockholm University, Stockholm, Sweden; 8 Institute of Climate and Energy Systems (ICE-3), Forschungszentrum Jülich GmbH, Jülich, Germany; 9 Institute of Astronomy, Geophysics and Atmospheric Sciences, University of São Paulo, São Paulo, Brazil; 10 Laboratoire d’Aérologie, Université de Toulouse, CNRS, UT3, IRD, Toulouse, France; 11 Flight Experiments, German Aerospace Center (DLR), Weßling, Germany; 12 Institute for Atmospheric Physics, Johannes Gutenberg-University, Mainz, Germany; 13 National Institute for Space Research, Cachoeira Paulista, Brazil; 14 National Institute of Amazonian Research, Manaus, Brazil; 15 Institute of Meteorology and Climate Research (IMK), Karlsruhe Institute of Technology (KIT), Eggenstein-Leopoldshafen, Germany; 16 Department of Geology and Geophysics, King Saud University, Riyadh, Saudi Arabia; 17 Scripps Institution of Oceanography, University of California, San Diego, La Jolla, CA USA; 18 Center for Sustainable Amazon Studies (CEAS), University of São Paulo, São Paulo, Brazil; 19 Instituto de Física, University of São Paulo, São Paulo, Brazil; 20 Climate and Atmosphere Research Center, The Cyprus Institute, Nicosia, Cyprus

**Keywords:** Atmospheric chemistry, Climate sciences, Atmospheric chemistry

Correction to: *Nature* 10.1038/s41586-024-08192-4 Published online 4 December 2024

In the version of this article initially published, there was a typographical error in the units shown on the Fig. 3b *y* axis, where “C* (μg m^–3^)” originally read “C* (μg cm^–3^).” In the article, the original text “Saturation vapour pressure C*” has been amended to read “Saturation concentration C*” in the Fig. 3b caption, in the last paragraph of the “Formation of organonitrates” section, and in four places in the now edited “Saturation concentration” Methods subsection. This correction does not influence our results and conclusions. The figure and text have been amended in the HTML and PDF versions of the article. We thank Dr. John Ness for alerting us to the need for this clarification.

